# Suppressive effect of exogenous carbon monoxide on endotoxin-stimulated platelet over-activation via the glycoprotein-mediated PI3K-Akt-GSK3β pathway

**DOI:** 10.1038/srep23653

**Published:** 2016-03-29

**Authors:** Dadong Liu, Xu Wang, Weiting Qin, Jingjia Chen, Yawei Wang, Mingfeng Zhuang, Bingwei Sun

**Affiliations:** 1 Department of Critical Care Medicine, Affiliated Hospital, Jiangsu University, Zhenjiang, Jiangsu Province, China; 2 Department of Burns and Plastic Surgery, Affiliated Hospital, Jiangsu University, Zhenjiang, Jiangsu Province, China; 3 School of Science, Jiangsu University, Zhenjiang, Jiangsu Province, China

## Abstract

Platelet activation is an important event involved in the pathophysiological processes of the coagulation system. Clinical evidence has shown that platelets undergo distinctive pathological processes during sepsis. Unfortunately, how platelets physiologically respond to inflammation or sepsis is not well understood. In this study, we used a lipopolysaccharide (LPS)-stimulated platelet model to systemically investigate alterations in membrane glycoprotein expression, molecular signaling, morphology and critical functions of platelets. We found that platelet adhesion, aggregation, secretion, and spreading on immobilized fibrinogen and the expression of platelet membrane glycoproteins were significantly increased by LPS stimulation, and these changes were accompanied by a significant decrease in cGMP levels and an abnormal distribution of platelet α-granules. Exogenous CO reversed these alterations. Profound morphological changes in LPS-stimulated platelets were observed using atomic force microscopy and phase microscopy. Furthermore, the elevated activities of PI3Ks, AKt and GSK-3β were effectively suppressed by exogenous CO, leading to the improvement of platelet function. Together, these results provide evidence that platelet over-activation persists under LPS-stimulation and that exogenous CO plays an important role in suppressing platelet activation via the glycoprotein-mediated PI3K-Akt-GSK3β pathway.

Platelet activation is an important event and is involved in the pathophysiological processes of the coagulation system. Emerging evidence suggests that activated platelets may play critical roles in many disease-related events, such as immune responses^1^, carcinogenesis^1^,^2^ and inflammatory responses^3^. However, the pathophysiological changes in platelets during sepsis are not well understood.

Sepsis, a systemic inflammatory response caused by severe systemic infection, continues to be a leading cause of morbidity and mortality^4^,^5^,^6^. It has been reported that LPS and inflammatory cytokines (e.g., tumor necrosis factor, TNF-α) potentiate the platelet activation that contributes to microthrombi formation in capillaries^6^,^7^. The important factors released from activated platelets, such as interleukin (IL) 1-β, monocyte chemoattractant factor (MCP-1) and platelet factor 4 (PF4), also play key roles in regulating inflammation and immune function^1^,^8^. In addition, many receptors in platelet membranes (e.g., glycoproteins) and molecular signaling molecules contribute to platelet activity and play an important role in the development of sepsis^9^,^10^,^11^,^12^. Therefore, clarifying the pathophysiological changes that occur in platelets during sepsis is essential to establishing novel therapeutic strategies.

It is well known that small amounts of CO are continuously produced in mammals, and the intracellular levels of this gaseous molecule markedly increase under stress conditions^13^,^14^. Studies have determined that exogenously administered CO has important cyto-protective functions and anti-inflammatory properties^15^,^16^,^17^,^18^. Recently, transition metal carbonyls have been identified as potential CO-releasing molecules (CORMs), which have a potential to facilitate the pharmaceutical use of CO by delivering it to the affected tissues and organs^13^,^19^. Studies have also shown that CORM-2 suppresses LPS-induced inflammatory responses *in vitro*^20^,^21^. Similarly, many studies have confirmed that this exogenous CO can rescue mice from lethal endotoxemia and sepsis induced by LPS or cecal ligation and puncture (CLP)^22^,^23^,^24^,^25^,^26^. Our previous studies have shown that CORM-2 inhibits the over expression of adhesion molecules, attenuates leukocyte sequestration in the organs of mice with CLP- or burn-induced sepsis, and decreases intracellular oxidative stress and NO production in LPS-stimulated HUVECs^27^,^28^,^29^,^30^. However, no studies have assessed the effects of exogenous CO on its regulation of platelet activation and the interactions between inflammation and coagulation during sepsis.

On the basis of the above information, the present study was designed as a prospective experiment to investigate the effects of exogenous CO on the suppression of LPS-stimulated platelet activation and to explore whether the molecular mechanisms of this therapeutic effect of CO occur via the glycoprotein-mediated PI3K-Akt-GSK3β pathway.

## Materials and Methods

Detailed descriptions of the materials and methods are available in the Online Data Supplement (Additional file). A brief description of the methods used is given below.

### Ethics statement

The Medical Ethical Committee of Jiangsu University approved the study. After written informed consent, blood specimens were extracted from healthy drug-free donors’ cubital veins. Consent for the use of these samples was given by the Medical Ethical Committee of Jiangsu University. All experiments were performed in accordance with the approved guidelines.

### LPS-stimulated platelet model

Blood was obtained from healthy volunteers. Platelet-rich plasma (PRP) and platelet-poor plasma (PPP) were obtained as described previously^31^.

LPS (10 μg/mL) was used to stimulate PRP and induce platelet activation. CORM-2 (Supplementary Figure 2) and iCORM-2 (an inactive form of CORM-2) were administered at different concentrations as a potential therapeutic agents. The different experimental PRP treatments were randomly assigned to five groups. The control group did not undergo any treatment, the LPS group received LPS (10 μg/mL) treatment for 30 min, and the CORM-2 group and iCORM-2 group underwent the same stimulation of LPS plus immediate administration of CORM-2 (10 or 50 μM) and iCORM-2 (50 μM). Additional experiments with CORM-2 pre-conditioning or delayed treatment were also performed. In some experiments, molecular signaling inhibitors for PI3K (LY294002), Akt (SH-6) and GSK-3β (CHIR99021) were incubated with the platelets for 10 min before LPS stimulation.

### Platelet adhesion, aggregation, spreading and secretion

Platelet adhesion, aggregation and spreading were measured as described previously^31^. Dense granule secretion was determined by measuring adenosine triphosphate (ATP) release as previous described^32^. P-selectin exposure was examined by flow cytometry to determine α-granule secretion^33^.

### Flow cytometry

Samples were collected and fixed in 1% paraformaldehyde for 15 min at room temperature followed by an incubation with FITC-labeled CD41 (CD41-FITC). Primary antibodies specific to P-selectin-PE, GPVI-eFluor 660, GPIbα-PE and integrin β_3_-PE were independently added into the samples, and IgG1-eFluor 660 and IgG1-PE were applied as isotype control antibodies. All samples were incubated in the dark for 30 min at room temperature, washed three times and then analyzed via flow cytometry^34^.

### Immunofluorescence

For the visualization of granule distribution, washed platelets were fixed, permeabilized, blocked and incubated with the corresponding antibodies overnight as previously described^34^. An Olympus IX71 fluorescence microscope (Olympus, Japan) was used to capture the images. All images were processed using cellSens Standard 1.12 software.

### Electron microscopy

Morphological evaluation of the platelets was conducted with a scanning electron microscope (LEO 440, Leica and Zeiss Co., Cambridge, England) or a transmission electron microscope (TEM) (JEM-2100, Japan Electronics Co., Ltd.) as previously described^35^.

### Atomic force microscopy (AFM) imaging

The stimulated platelets were prepared as describes above. AFM was used to acquire topographic and phase images of platelets and to determine the roughness of the cell membranes with a tapping mode in an atmospheric environment. The procedure was repeated for five cells, and each cell was scanned at least three times. After imaging the whole cells, the adhesive force of the cell membranes was further assayed with AFM in a force-modulated mode, and the force-distance curves were drawn. All force–distance experiments were performed at the same loading rate. Adhesive forces were obtained using the contact process between the probe tip and the sample surface^36^,^37^,^38^.

### Phase microscopy methods

Platelets were also observed with a BioPhase device. BioPhase is a new imaging device based on Digital Wavefront Technology that performs real time cellular imaging using standard optical microscopes. When light waves travel through the transparent biological specimens, the phase of the transmitted wave changes in a manner dependent on the properties of the specimens. The changes of phase can be determined with a phase microscopy technique, which reveals information about intracellular and extracellular structures^39^. With this technique, the volume, shape and surface roughness of intracellular structures, primarily organelles, can be determined^40^,^41^. Briefly, the stimulated platelets were prepared as described above. Then, samples were fixed by mixing them with an equal volume of 1% paraformaldehyde for 15 min at room temperature. Finally, the platelets were visualized under an optical microscope (OLYMPUS GX51) using the BioPhase imaging device. All images were processed using MATLAB (r2014a) software.

### ELISA

Platelet cGMP accumulation was measured by using a standard ELISA kit as previous described^42^. Briefly, platelets were hydrolyzed with cell lysis buffer. The lysates were centrifuged and cGMP in supernatants was measured using cGMP ELISA Kit according to the manufacture’s protocol.

### Immunoprecipitation and western blot analysis

The platelets were processed with the addition of RIPA buffer containing a protease and phosphatase inhibitor cocktail. A portion of the lysate was collected via immunoprecipitation for the detection of target phosphorylated proteins. For the detection of target proteins, samples were processed via immunoblotting as described previously^42^. The bands were visualized with an ECL reagent and Hyper film ECL as described by the manufacturer. Films were scanned using a flatbed scanner, and the bands were quantified using Basic Quantifier software, an image analysis program.

### Statistics

All data are presented as the mean ± standard deviation. Statistical analyses were performed with a one-way analysis of variance (ANOVA) and a post hoc test (least significant difference, LSD test). A value of P < 0.05 was considered to be statistically significant. All analyses were performed using SPSS 16.0 (Chicago, IL, USA).

## Results

### Effects of CORM-2 on platelet adhesion, aggregation and spreading

**Figure 1:**
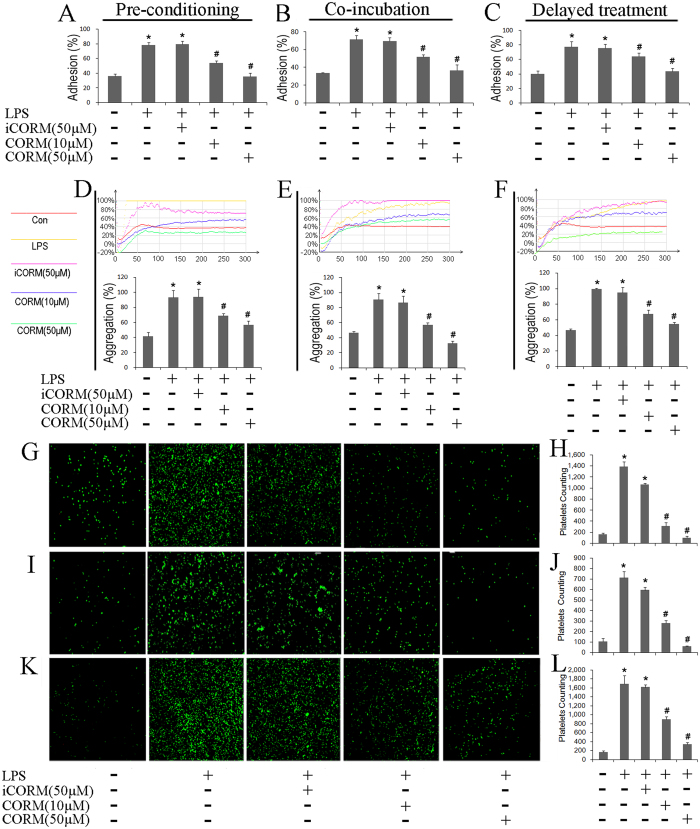
Effects of CORM-2 on LPS-stimulated platelet adhesion, aggregation and spreading. Platelets were stimulated with LPS (10 μg/ml) for 30 min in the presence or absence of CORM-2 (10, 50 μM) applied using a co-incubation, preconditioning or delayed treatment, as described in the “Materials and methods” section. After the 30-minute stimulation of LPS, a significant increase in platelet adhesion and aggregation was detectable. Treatment with CORM-2 (10, 50 μM) effectively abolished this increase in a concentration-dependent manner (**B**,**E**). LPS stimulation resulted in a significant increase in platelet spreading on immobilized fibrinogen. However, the treatment of platelets with CORM-2 significantly abolished this spreading (**I**,**J**). Similar results were also detected in the CORM-2 pre-conditioned (**A**,**D**,**G**,**H**) and CORM-2 delayed-treatment groups (**C**,**F**,**K**,**L**). The results are presented as the mean ± SE of five experiments. *P < 0.01 compared with the control, ^#^P < 0.05 compared with the LPS treatment. Note that LPS-induced platelet aggregation and spreading were inhibited by CORM-2 in a dose-dependent manner.

In the present experiments, different functions of LPS-stimulated platelets were detected. After a 30-minute stimulation of platelets with LPS, a significant increase in platelet adhesion was detected. Treatment with CORM-2 (10, 50 μM) effectively abolished this increase in a concentration-dependent manner (Fig. 1B). A similar alteration in aggregation was also observed (Fig. 1E). To determine whether LPS influenced platelet spreading, platelets were stimulated with LPS for 30 minutes and then allowed to spread on a fibrinogen-coated surface. We found that LPS stimulation resulted in a significant increase in platelet spreading on immobilized fibrinogen. However, the treatment of platelets with CORM-2 significantly abolished this platelet spreading (Fig. 1I,J). Surprisingly, similar results were also detected in the CORM-2 pre-conditioned (Fig. 1A,D,G,H) and CORM-2 delayed-treatment groups (Fig. 1C,F,K,L). Hence, these data indicate that LPS stimulation can significantly influence the ability of platelets to increase their adhesion, aggregation and spreading, leading to platelet activation. Exogenous CO may improve platelet function and then inhibit platelet activation.

### Effects of CORM-2 on platelet secretion and α-granule distribution

**Figure 2:**
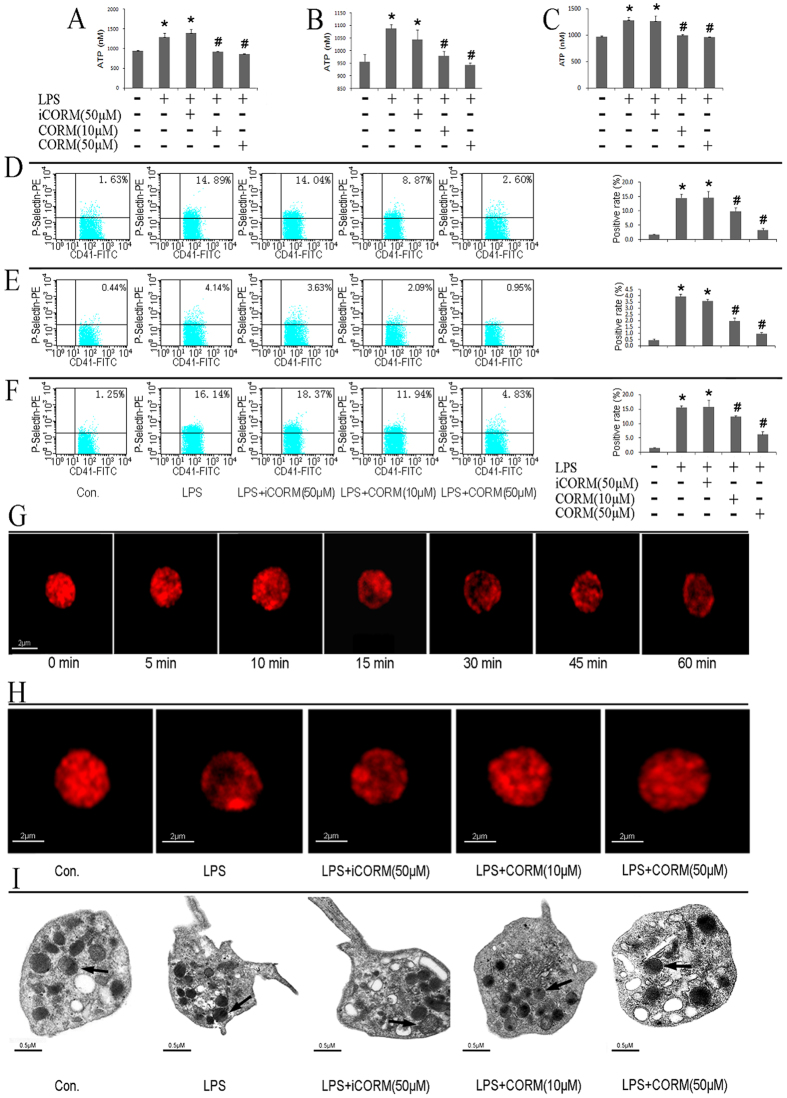
Effects of CORM-2 on secretion and α-granule distribution in LPS-stimulated platelets. Platelets were stimulated and treated as described in Fig. 1. LPS stimulation resulted in a significant increase in ATP release from platelets. Treatment of platelets with CORM-2 significantly reduced the ATP release in response to LPS stimulation (**B**). Similar results were found in the CORM-2 pre-conditioned (**A**) and delayed-treatment groups (**C**). P-selectin expression was detected via flow cytometry. The results indicate that LPS stimulation resulted in a significant increase in P-selectin expression, whereas treatment with CORM-2 significantly reduced this increase (**E**). Similar results were found in the CORM-2 pre-conditioned **(D**) and delayed-treatment groups (**F**). Samples were fixed for transmission electron microscopy (TEM) and immunofluorescence analysis. Platelets were labeled with antibodies to VAMP8 followed by staining with secondary antibodies labeled with Alexa Fluor 594. Images were taken at the indicated times. A series of representative images demonstrate LPS-induced platelet α-granule distribution at different time points: 0 min, 5 min, 10 min, 15 min, 30 min, 45 min, and 60 min (**G**). Representative electron microscopy images (**I**) and immunofluorescence images (**H**) show clear changes in platelet α-granule distribution under LPS stimulation and co-incubation with iCORM-2 (50 μmol/L). After treatment with CORM-2 (10 or 50 μmol/L), the amount of α-granules fused with platelet membranes decreased. Representative α-granules are indicated by arrows. Scale bars indicate 0.5 μm for the electron microscopy images and 2 μm for the immunofluorescence images. The results are the mean ± SE (n = 5). *P < 0.01 compared with control, ^#^P < 0.05 compared with LPS.

Platelet activation results in the release of granule contents, and in turn, granule exocytosis and secretion may continue to potentiate the platelet activation induced even by the low-dose agonists^43^. Here, platelet secretion was examined by measuring the level of ATP release and P-selectin expression, which were quickly released from platelet α-granules and presented on the surface of the activated platelets. We found that LPS stimulation resulted in a significant increase in ATP release from platelets. However, the treatment of platelets with CORM-2 significantly reduced the amount of ATP released in response to LPS stimulation (Fig. 2B). Similar results were found in relation to P-selectin expression (Fig. 2E) and in the CORM-2 pre-conditioned (Fig. 2A,D) and CORM-2 delayed-treatment groups (Fig. 2C,F).

In addition, immunofluorescence images show that the distribution of platelet α-granules labeled with antibodies to VAMP8 was clearly changed after LPS stimulation (5 min) and that platelet α-granules moved from the central granulomere to the platelet plasma membrane at the indicated times (Fig. 2G). These changes included plasma membrane ruffling, irregularities, and extensions and an acceleration in the rates of α-granule membranes fusing with platelet membranes and α-granule exocytosis. After CORM-2 treatment for 30 min, the assembly of the platelet α-granules at the platelet plasma membranes was significantly decreased (Fig. 2H, Supplementary Figure 2A). In parallel, changes in the distribution of the platelet α-granules detected using immunofluorescence were consistent with the TEM results (Fig. 2I).

### Effects of CORM-2 on morphological changes in platelets and platelet phase shifts

**Figure 3:**
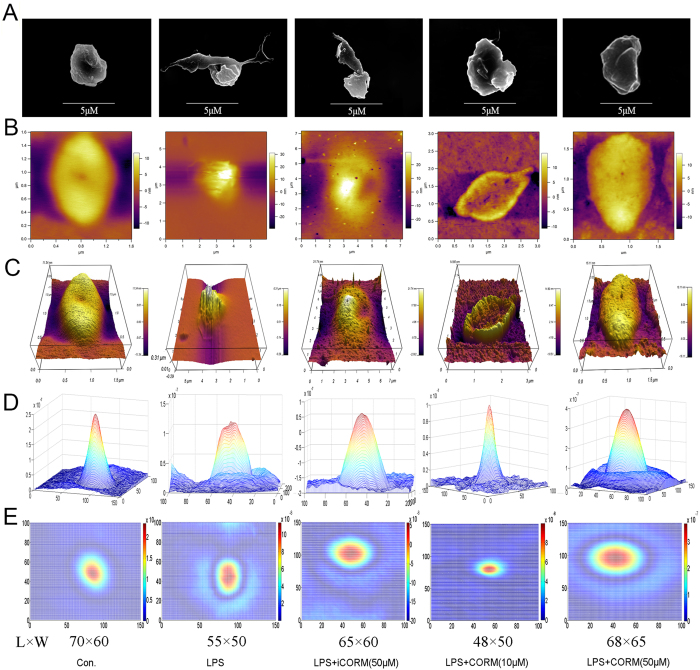
Effects of CORM-2 on LPS-stimulated changes in platelet morphology and platelet phase shifts. Platelets were stimulated with LPS and treated with CORM-2 as described in Fig. 1. Scanning electron microscopy (SEM) was used to assess LPS-stimulated changes in platelet morphology. After LPS stimulation, the shapes of platelets became irregular with rough surfaces and pseudopodia. Similar results were also observed in the iCORM-2 group. However, co-incubation with CORM-2 markedly reversed the deformation of platelets, and the generation of pseudopodia was also suppressed (**A**). The topographic and phase images of platelets acquired using AFM (**B,C**). 3D phase-rendered images of platelets are shown in (**D**), and the top view of the 3D phase images of platelets are shown in (**E**). Images correspond to the control, LPS, LPS+iCORM-2, LPS+CORM-2 (10 μM), LPS+CORM-2 (50 μM) groups, respectively. Color bar, phase at λ = 633 nm. L × W: length × width = the product of the lattice numbers of the length and width of each piece in the area with color differences, which indirectly reflects the cell size.

The release of granule contents described above resulting from platelet activation was accompanied by profound morphological changes. To assess the morphological changes in LPS-stimulated platelets, scanning electron microscopy (SEM) was used. As shown in Fig. 3(A), the shapes of platelets became irregular, and they had rough surfaces and pseudopodia after LPS stimulation. However, co-incubation with LPS and CORM-2 markedly reduced the deformation of platelets, and the generation of pseudopodia was also suppressed. The topographic and 3D images of platelets were acquired using AFM (Fig. 3B,C). The average roughness (Ra) and root mean square roughness (Rq) were calculated to determine the roughness of the cell membranes. As shown in Supplementary Figure 3A,B, the Ra and Rq values were significantly increased in the LPS-challenged platelets, whereas the increase was inhibited in the CORM-2-treated groups. The height profiles of the platelets were also observed. The results show that after LPS stimulation, the height curve of the platelets manifested as a steep peak with an increased average height. However, co-incubation with CORM-2 significantly reversed the effects on the height profile and resulted in dual peaks with a rough curve (Supplementary Figure 3C,E). Force-distance information was acquired to determine the adhesive forces of the cell membranes. As shown in Supplementary Figure 3D,F, adhesive forces increased after LPS stimulation but were attenuated in a dose-dependent manner when platelets were treated with CORM-2.

As shown in Fig. 3D,E and Supplementary Figure 2B, the height and color of a point on the image correspond to the platelet’s thickness and depend only on the relative refractive index. The platelets in the control group were found to be spherical because they appeared to have raised centers. After being stimulated by LPS, each formerly raised center was extended toward its two ends. Under the assumption that the platelet cytoplasm was homogeneous, this change was induced by organelles (e.g., granules or mitochondria) with a high thickness and relative refractive index distributed toward both ends (membranes) of the platelets. Similar results were observed in the iCORM-2 group. However, co-incubation with LPS and CORM-2 markedly reduced the change described above, and the raised center did not extend toward its two ends.

### Effects of CORM-2 on expression of membrane glycoproteins

**Figure 4:**
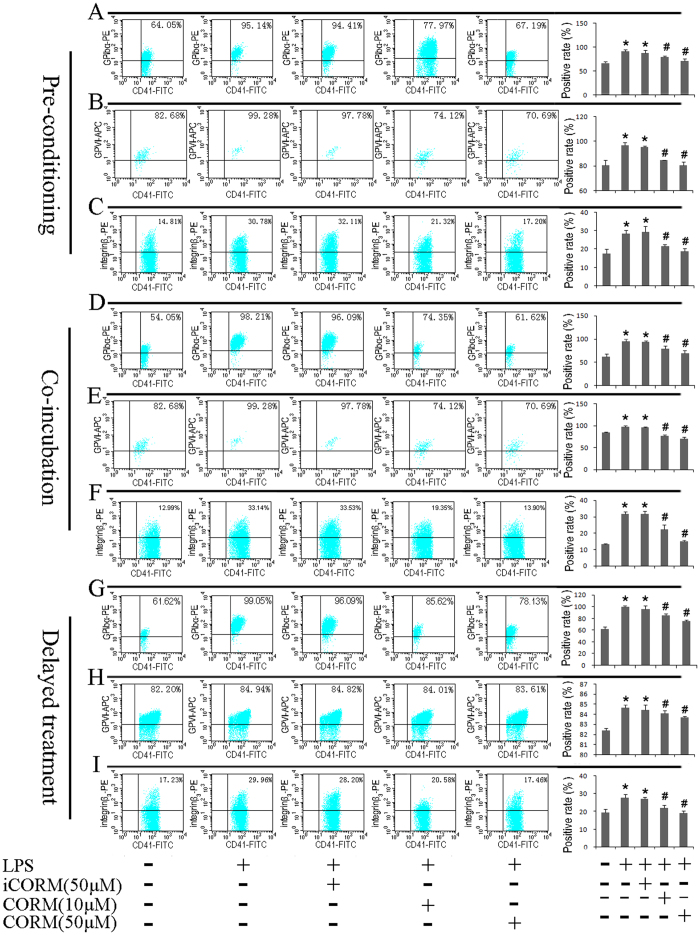
Effects of CORM-2 on the expression of membrane glycoproteins in LPS-stimulated platelets. Platelets were stimulated by LPS and treated with CORM-2 as described in Fig. 1. All samples were incubated in the dark for 30 min, washed three times and analyzed via flow cytometry. The expression of membrane glycoproteins (GPIbα, GPVI and GPIIb/IIIa)) was significantly upregulated in LPS-stimulated platelets, whereas exogenous CO administration inhibited this upregulation (**D**–**F**). Similar results were also observed in the CORM-2 pre-conditioned (**A**–**C**) and delayed-treatment groups (**G**–**I**). The results are shown as the mean ± SE (n = 5). *P < 0.01 compared with the control, ^#^P < 0.05 compared with the LPS treatment. Note that LPS-induced membrane glycoprotein expression was downregulated by CORM-2 in a dose-dependent manner.

Previous studies have suggested that platelet spreading on immobilized fibrinogen is dependent on cytoskeletal reorganization driven by outside-in signaling mediated by glycoproteins such as GPIbα and GPVI^9^,^44^. In this experiment, the expression of important membrane glycoproteins (GPIbα, GPVI and integrinβ_3_) in LPS-stimulated platelets was measured via flow cytometry. The results show that the expression of membrane glycoproteins was significantly upregulated in LPS-stimulated platelets, whereas exogenous CO administration inhibited this upregulation (Fig. 4D–F). Interestingly, similar results were also observed in the CORM-2 pre-conditioned (Fig. 4A–C) and CORM-2 delayed-treatment groups (Fig. 4G–I). Combined with the results presented above, these data indicate that exogenous CO may affect glycoprotein-mediated outside-in signaling, resulting in improvements in platelet function during LPS stimulation.

In particular, GPIIb/IIIa (also named integrin α_IIb_β_3_), the most abundant platelet surface protein, and one that is required for various hemostatic functions, was found to be regulated by inhibitors of signal molecules (PI3K, Akt and GSK-3β). Integrinβ_3_ is the functional subunit of integrin α_IIb_β_3_ and is responsible for key signal transduction steps. As mentioned above, integrinβ_3_ was significantly upregulated in LPS-stimulated platelets. However, this upregulation was effectively inhibited by inhibitors of PI3K (LY294002), Akt (SH-6) and GSK-3β (CHIR99021), as well as by CORM-2 (Supplementary Figure 4). These interesting results show that GPIIb/IIIa (at least integrinβ_3_) is an important target regulated by molecular signaling proteins, and they reveal that exogenous CO acts similarly to a signal molecule inhibitor in the regulation of GPIIb/IIIa expression. These results imply that exogenous CO may be a multi-targeting compound with a variety of means to regulate signal transmission in intervention treatments.

### Effects of CORM-2 on cGMP secretion and signal molecule expression/phosphorylation

**Figure 5:**
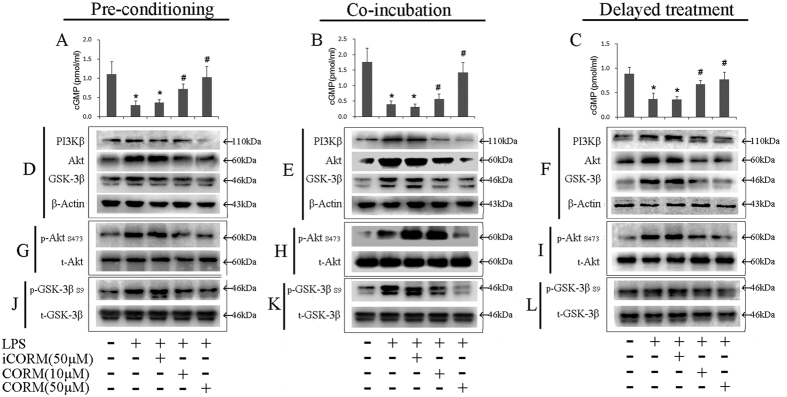
Effects of CORM-2 on cGMP secretion and signal molecule expression/phosphorylation in LPS-stimulated platelets. Platelets were stimulated by LPS and treated with CORM-2 as described in Fig. 1. Platelet cGMP accumulation was measured using a standard ELISA kit. LPS stimulation resulted in a significant decrease in platelet cGMP, whereas CORM-2 treatment in LPS-stimulated platelets significantly abolished this decrease in a concentration-dependent manner in the CORM-2 pre-conditioned (**A**), co-incubation (**B**) and delayed-treatment groups (**C**). Platelets were lysed in RIPA buffer containing protease and phosphatase inhibitor cocktails, and signal molecule expression and phosphorylation were assessed. LPS stimulation resulted in a significant increase in the PI3Kβ level, and this increase was abolished in LPS-stimulated platelets treated with CORM-2 (**E**). After treatment with exogenous CO, Akt production (**H**) and phosphorylation (**N**) in LPS-stimulated platelets were markedly inhibited. The same results were found for GSK-3β production and phosphorylation (**K,Q**). Similar results were also found in the CORM-2 pre-conditioned (**D**,**G**,**J**,**M**,**P**) and delayed-treatment groups (**F**,**I**,**L**,**O**,**R**). Representative experiments are shown in panels (**D–R**). The average ratios of the level of protein expression to phosphorylation are shown in Supplementary Figure 3. The results are presented as the mean ± SE (n = 5). *P < 0.01 compared with the control, ^#^P < 0.05 compared with the LPS treatment. Note that LPS-induced signal molecule expression and phosphorylation were downregulated by CORM-2 in a dose-dependent manner.

cGMP is generated from guanosine 5′-triphosphate (GTP)^45^,^46^. As a multi-functional messenger molecule, cGMP affects cellular activities through different pathways involving cGMP-dependent protein kinases (PKG), cyclic nucleotide-gated (CNG) channels, and phosphodiesterases^47^. To determine the effect of LPS stimulation on cGMP production, the levels of cGMP were examined via ELISA. We found that LPS stimulation resulted in a significant decrease in cGMP levels, whereas CORM-2 treatment of LPS-stimulated platelets significantly abolished this decrease in a concentration-dependent manner for all types of CORM-2 treatment: CORM-2 pre-conditioning (Fig. 5A), CORM-2 co-incubation (Fig. 5B) and CORM-2 delayed-treatment (Fig. 5C). These results indicate that cGMP level plays an important role in maintaining platelet function. The decrease of cGMP is relevant to platelet activation during LPS stimulation. Administration of exogenous CO may effectively elevate cGMP levels in LPS-stimulated platelets, resulting in a suppression of platelet activation.

To determine the effects of LPS on PI3K activity, PI3Kβ, which plays a predominant role in platelet function, was examined in LPS-stimulated platelets. We found that LPS stimulation resulted in a significant increase in the levels of PI3Kβ and that this increase was abolished in LPS-stimulated platelets treated with CORM-2 (Fig. 5E and Supplementary Figure 5B). Further studies revealed that treatment with exogenous CO markedly inhibited Akt production (Fig. 5E and Supplementary Figure 5E) and phosphorylation (Fig. 5H and Supplementary Figure 5K) in LPS-stimulated platelets. Interestingly, the same results were observed in relation to GSK-3β production and phosphorylation (Fig. 5E,K and Supplementary Figure 5H,N). As an important regulator, GSK-3β plays a key role in platelet activation by blocking GPIIbIIIa-mediated outside-in signaling^48^. LPS stimulation elevates GSK-3β activity in platelets, resulting in platelet activation. Exogenous CO effectively improves platelet function by suppressing GSK-3β activity in LPS-stimulated platelets. Notably, similar results were also found in the CORM-2 pre-conditioned (Fig. 5D,G,J and Supplementary Figure 5A,D,G,J,M) and CORM-2 delayed-treatment groups (Fig. 5F,I,L and Supplementary Figure 5C,F,I,L,O). These results suggest that LPS-induced platelet activation may result from an elevation of the activities of PI3Ks, AKt and GSK-3β and that the PI3K-dependent pathway may be one of the targets through which exogenous CO affects platelet function.

### Effects of CORM-2 and inhibitors on signal molecule expression/phosphorylation

**Figure 6:**
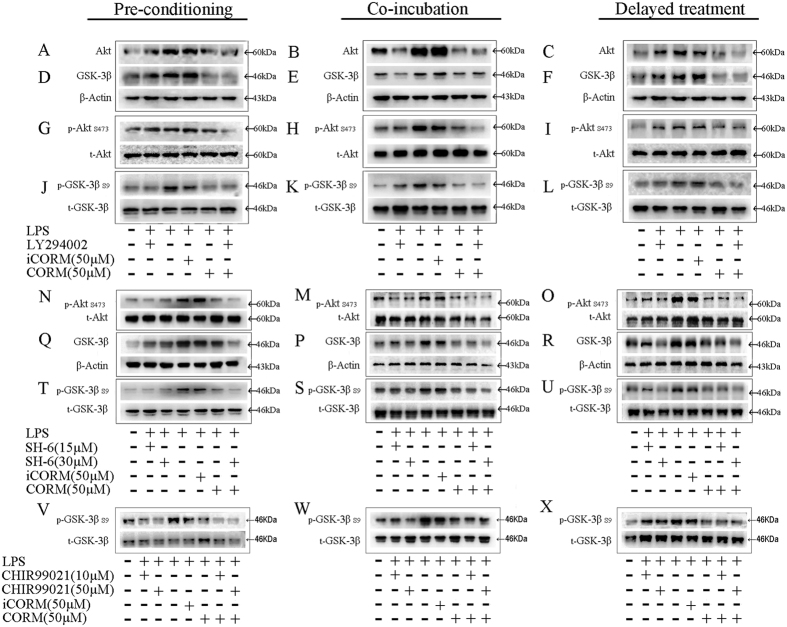
Effects of CORM-2 and signal molecular inhibitor in LPS-stimulated platelet. Platelets were stimulated by LPS and treated with CORM-2 as described in Fig. 1. The platelets were lysed in RIPA buffer that contained protease and phosphatase inhibitor cocktails. Signal molecular expression and phosphorylation were assessed in LPS-stimulated platelets with treatment of CORM-2 or signal molecular inhibitors. The increase of Akt production and phosphorylation were abolished simultaneously in LPS-stimulated platelet treated with LY294002, SH-6 or CORM-2 (**B,H,M**). Meanwhile, GSK-3β expression and phosphorylation were inhibited in LPS-stimulated platelet with administration of LY294002, SH-6 or CORM-2 (**E,K,P,S**), and no differences were observed between the LY294002, SH-6 and CORM-2 (50 μM) group. Also, GSK-3β phosphorylation inhibitor (CHIR99021) were used in the further exploration. The increase of GSK-3β phosphorylation was inhibited in LPS-stimulated platelet treated with CHIR99021 (**W**). The similar phenomenon was observed in the CORM-2 groups with or without CHIR99021. No differences among the CHIR99021 and CORM-2 groups were observed. Similar results were also shown in CORM-2 pre-conditioning (**A,G,N,D,J,Q,T,V**) or CORM-2 delayed treatment group (**C,I,O,F,L,R,U,X**). Representative experiments are shown in (**A**–**X**). Quantitative analysis of protein expression and phosphorylation level was shown in Supplementary Figures 4 and 5.

As described above, we found that exogenous CO may effectively abolish the significant elevations in PI3K, Akt and GSK-3β production and phosphorylation in LPS-stimulated platelets. Further studies were conducted with application of the signal molecule inhibitors LY294002, SH-6 and CHIR99021, which are selective inhibitors of PI3K, Akt and GSK-3β, respectively, to explore whether exogenous CO plays the same role as the inhibitors or whether they have synergistic actions. Interestingly we found that the increases in Akt production and phosphorylation were simultaneously abolished in LPS-stimulated platelets treated with LY294002, SH-6 or CORM-2 (Fig. 6B,H,M; Supplementary Figure 6B,H; and Supplementary Figure 7B). Moreover, GSK-3β expression and phosphorylation were inhibited in LPS-stimulated platelets by the administration of LY294002, SH-6 or CORM-2 (Fig. 6E,K,P,S; Supplementary Figure 6E,K; and Supplementary Figure 7E,H), and no differences were observed between the LY294002, SH-6 and CORM-2 groups. These results reveal that inhibition of PI3K activity results in the secondary inhibition of Akt activity and subsequently suppresses GSK-3β expression and phosphorylation.

To better clarify the effect of GSK-3β on platelet activation, a GSK-3β phosphorylation inhibitor (CHIR99021) was used in further experiments. As expected, we found that the increase in GSK-3β phosphorylation was inhibited in LPS-stimulated platelets treated with CHIR99021 (Fig. 6W and Supplementary Figure 7K). A similar phenomenon was observed in the CORM-2 groups with or without CHIR99021. Likewise, no differences among the CHIR99021 and CORM-2 groups were observed. Similar results were also shown in the CORM-2 pre-conditioned (Fig. 6A,G,N,D,J,Q,T,V and Supplementary Figure 7J) and CORM-2 delayed-treatment groups (Fig. 6C,I,O,F,L,R,U,X and Supplementary Figure 7L).

## Discussion

Accumulating clinical evidence shows that, during sepsis, platelets undergo distinctive pathological processes that are closely related to the general condition of the patient^1^,^49^,^50^. Unfortunately, how platelets physiologically respond to inflammation or sepsis is not well known. An incomplete understanding of abnormal platelet activation in sepsis results in insufficient therapeutic strategies, thus leading to persistently high mortality rates among septic patients. Therefore, more effective strategies directed against abnormal platelet activation should be developed and included in new guidelines for therapeutic strategies in treating sepsis. In this study, we used an LPS-stimulated platelet model to systemically investigate alterations in the expression of platelet membrane glycoproteins and key signal molecules, and the morphology and critical function of platelets. Based on the results we achieved previously^13^,^27^,^28^, CORM-released CO, acting as exogenous CO, was used in this study as a therapeutic strategy to explore whether exogenous CO can suppress platelet over-activation and to examine the possible underlying mechanisms for this over-activation.

The biological mechanisms of platelet activation have been well delineated in pathological bleeding and thrombosis, whereas the special variations of platelet function during sepsis have not been clarified. In this experiment, we found that the key functions of platelets, such as adhesion, aggregation, spreading and secretion, were enhanced after LPS stimulation, whereas CORM-2 significantly suppressed these increases regardless of whether it was used as a pre-conditioning or delayed treatment. Hence, these data indicate that exogenous CO may be a key factor for decreasing platelet function and inhibiting platelet activation.

A change in platelet morphology is another key mechanism for the maintenance of platelet function. In LPS-stimulated platelets, clear morphological changes were observed, such as irregular oval shapes, the formation of filopodia and the centralization of organelles. Under SEM, the shape of platelets became irregular, with rough surfaces and pseudopodia appearing after LPS stimulation. However, treatment with CORM-2 markedly reversed the deformation of platelets, and the generation of pseudopodia was also suppressed. Additionally, AFM was used to evaluate the morphological changes in the platelets. AFM imaging and quantitative determinations were carried out with a cantilever by sensing and amplifying the contact force between the probe and the sample atom. AFM, which provides atomic-level resolution, has broad applications in biological, food and medical research. AFM allows for precise observations of the cell surface and quantitative assessments of superficial area, thickness, width and volume to achieve a three-dimensional diagram. Additionally, cell micro-mechanical properties and a force curve can be obtained via AFM^51^,^52^,^53^,^54^. In this study, platelets were stimulated with LPS in the presence or absence of CORM-2, and then AFM was used to acquire topographic and phase images of platelets by using a tapping mode. The roughness of the cell membranes was also detected, and the results are expressed as Rq and Ra. The data show that the shape of the platelets became irregular and developed a rough surface (the values of Rq and Ra were increased) and pseudopodia in the LPS-treated group. However, CORM-2 significantly reversed the LPS-stimulated changes in platelet morphology. The adhesive force of the cell membrane was further assayed by using AFM, and force-distance curves were drawn. The information acquired from the adhesive force tests implied that LPS stimulation increased the adhesive force of the cell membranes and that this increase was abolished by the presence of CORM-2.

To better describe the changes in platelet morphology and structure, phase microscopy was used in this study. Because it is non-invasive, non-damaging and provides quantifiable images, phase microscopy has become a very effective tool among the many techniques available for the biological study of cells^55^,^56^. There are different refractive indexes for the cells and the surrounding medium, which result in different phase shifts when light passes through them. The phase shifts contain information about the refractive index distribution and morphology of the cells. In this study, platelets were co-incubated with LPS and CORM-2 and phase microscopy was used to obtain the phase shifts of the platelets. The information acquired from the phase images implies that the raised center was extended toward the two ends of the platelets in the LPS-treated group. CORM-2 attenuated these changes, which made the phase image look more like that of the control group, without any extensions towards the ends. This result is consistent with the findings of the SEM or AFM analyses discussed above. Thus, we suggest that CORM-2 can systematically contribute to the maintenance of normal platelet morphology and the inhibition of platelet activation. What then, is the mechanism by which exogenous CO therapeutically activates platelets?

A large body of evidence has demonstrated that platelet receptors, upon receiving a stimulus, transmit signals via their platelet activation pathway and ultimately induce the “inside-out” signaling process that leads to integrin activation^9^,^57^. Among these receptors, the GPIb-V-IX complex and GPVI play major roles in platelet activation. The GPIb-V-IX complex is a heteromeric complex constitutively expressed on the platelet membrane and includes four different polypeptide chains. GPIbα is the major receptor of GPIb-V-IX and plays a role in many aspects of platelet function, particularly in platelet activation by von Willebrand factor (vWF)^7^,^58^,^59^. GPVI is a type I transmembrane glycoprotein of the immune-receptor family with two extracellular immunoglobulin domains, a mucin domain and a transmembrane domain, and it has a cytoplasmic tail that leads to platelet activation via the attached collagen^9^,^59^,^60^,^61^. In this study, we found that the expression of both membrane GPIbα and GPVI in LPS-stimulated platelets was significantly upregulated. This phenomenon was effectively inhibited by the administration of CORM-2. Interestingly, the LPS-induced upregulation of membrane glycoproteins was inhibited in the present study by both CORM-2 preconditioning and delayed treatment. In addition, a key event in the process of platelet activation is the activation of GPIIb/IIIa, which is thought to be an anti-thrombotic target that bridges adjacent platelets and mediates stable platelet adhesion to the ECM by binding to fibrinogen, fibronectin, von Willebrand factor (vWF) and multiple ECM proteins^62^,^63^. The conformational change of GPIIb/IIIa may induce further signaling, promoting actin polymerization and cytoskeletal reorganization in a process termed outside-in signaling^64^. The observations made in the present study suggest that the inhibition of GSK-3β activity results in a suppression of platelet activation by blocking GPIIb/IIIa overexpression. Our work further suggests that exogenous CO and various inhibitors (LY294002, SH-6 and CHIR99021) decrease the expression of GPIIb/IIIa in LPS-induced platelet activation. Similar results were found with the use of CORM-2 pre-conditioning or CORM-2 delayed treatment. From these results, we suggest that the most important potential mechanism by which exogenous CO inhibits platelet activation is through its prevention of the over-expression of membrane glycoproteins, which are the therapeutic target for platelets, which then affects membrane glycoprotein-mediated outside-in signaling.

It is well known that the PI3K signaling pathway is important to many aspects of cell growth, migration, and survival^65^,^66^. Human and mouse platelets express all class I PI3K isoforms (PI3Kα, PI3Kβ, PI3Kδ, and PI3Kλ)^10^,^67^. Class I PI3Ks are primarily involved in transmitting signals from membrane receptors and are composed of heterodimers of an inhibitory adaptor/regulatory subunit (p85) and a catalytic (p110) subunit^82,85^. The p85 subunit binds and integrates signals from various cellular proteins. The phosphorylated p85 subunit facilitates the full activation of the p110 catalytic subunit, which activates PI3K^48^,^66^,^68^. PI3K activation results in an increase in PI (3, 4, 5)P3 (PIP3), which recruits PH domain-containing kinase 1 (PDK1) and Akt to the membrane where PDK1 phosphorylates Akt on Thr^308^ in the T-loop. Akt is subsequently phosphorylated by mTORC_2_ on Ser^473^, resulting in maximal Akt activity^12^,^69^. The Akt family of serine/threonine kinases includes the Akt1, Akt2, and Akt3 isoforms that are expressed in platelets and plays an important role in platelet activation^70^,^71^,^72^. The PI3K/Akt pathway regulates many cellular processes, including metabolism, proliferation, survival, growth and angiogenesis^66^,^72^. In this experiment, we found that both the production and phosphorylation of PI3Kβ and Akt in LPS-stimulated platelets were significantly upregulated. Use of LY294002 and SH-6 to independently inhibit PI3K and Akt activity resulted in a significant downregulation of Akt production and phosphorylation in LPS-stimulated platelets. Similar results were shown in the CORM-2 pre-conditioned and delayed-treatment groups. These results strongly suggest that the PI3K/Akt pathway plays an important role in LPS-induced platelet activation. The administration of exogenous CO was capable of effectively affecting the PI3K/Akt pathway.

The Akt substrate, glycogen synthase kinase (GSK) phosphorylates and inhibits the activity of glycogen synthase^66^. GSK-3, a constitutively active serine-threonine kinase, has two isoforms, α and β. GSK-3β is expressed predominantly in platelets, and its activity is inhibited at Ser^9^ by Akt phosphorylation^66^,^73^. However, it is not known whether GSK-3β is a major target of the PI3K/AKT signaling pathway.

Li *et al.* have reported that GSK-3β^+/−^ platelets, compared with WT platelets, demonstrate enhanced agonist-dependent aggregation, dense granule secretion, and fibrinogen binding. Treatment of human platelets with GSK3 inhibitors renders them more sensitive to agonist-induced aggregation, suggesting that GSK3 suppresses platelet function *in vitro*. Therefore, the authors have concluded that GSK-3β acts as a negative regulator of platelet function and thrombosis *in vitro* and *in vivo*^73^. However, Barry *et al.* have reported that three structurally distinct GSK3 inhibitors, lithium, SB415286 and TDZD-8, inhibit platelet aggregation^74^. Another study has also indicated that the administration of a GSK3 inhibitor potently suppresses the proinflammatory response in mice treated with lipopolysaccharide, and mediates protection from endotoxin shock^75^. These reports are apparently paradoxical, but the findings presented in this study clearly support the latter conclusion. We found that the production and phosphorylation of GSK-3β in LPS-stimulated platelets was markedly increased. However, exogenous CO administration clearly inhibited GSK-3β phosphorylation, indicating that exogenous CO directly or indirectly inhibits GSK-3β activation. Moreover, the structure and function of platelets were both significantly improved via exogenous CO intervention. Similar results were also observed with the use of CHIR99021, a GSK-3β phosphorylation inhibitor. Further analysis showed that GSK-3β expression and its phosphorylation level were both effectively suppressed by a PI3K inhibitor (LY294002) or an Akt inhibitor (SH-6). These results were consistent with those from exogenous CO intervention, implying that CO plays an important role as a potential regulator of platelet activation. From these results, we suggest that the increase in GSK-3β phosphorylation after LPS stimulation markedly promotes platelet activation but that exogenous CO inhibits PI3K/Akt phosphorylation, leading to the suppression of GSK-3β phosphorylation and therefore the suppression of platelet activation.

It has been proposed that endogenous carbon monoxide functions as an endogenous messenger molecule and activates soluble guanylyl cyclase (sGC), thereby stimulating formation of cyclic guanosine 3′,5′-monophosphate (cGMP)^18^. Increases in intracellular cGMP, a second messenger, elicits upregulation of cGMP-dependent protein kinase type I and II (cGKI and II). cGKI lowers the intracellular level of cytosolic calcium and is therefore considered important for the inhibition of platelet activation. In this study, we found that treatment with exogenous CO elevated cGMP levels in LPS-stimulated platelets in a concentration-dependent manner. Moreover, improvements in platelet morphology and function reflected the original cGMP levels, thus suggesting that exogenous CO, acting through the cGMP pathway, may be a good candidate for potential therapeutic administration. These findings further support those from our previous study using other septic models, e.g., CLP- or burn-induced sepsis, which demonstrated that the administration of exogenous CO might be sufficient to maintain or upregulate the cGMP pathway, resulting in the inhibition of inflammatory responses and the protection of vital organ functions against sepsis.

**Figure 7:**
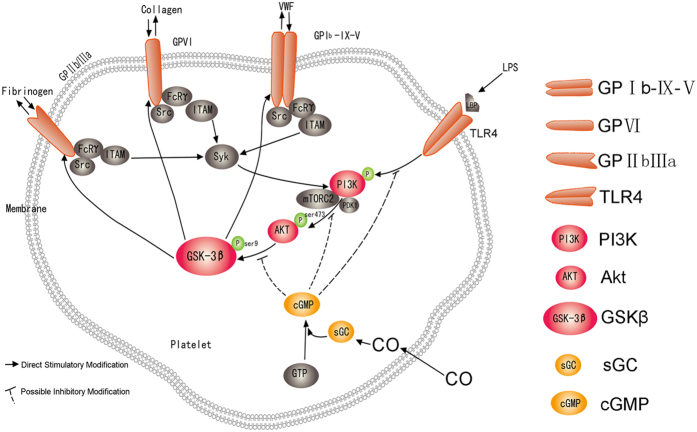
Mechanism of therapeutical strategy of exogenous carbon monoxide on platelet over activation via glycoprotein-mediated PI3K-Akt-GSK3 pathway.

In summary, data from the present study provide evidence that platelet activation persists under LPS stimulation. This study also provides a clear basis for the use of a novel anti-platelet agent, exogenous CO, as an effective strategy for improving platelet function and support, a model (Fig. 7) in which the glycoprotein-mediated PI3K-Akt-GSK-3β pathway effectively regulates platelet activation during sepsis. Undoubtedly, translating this understanding into the development of clinical treatments will be valuable. However, there is still a large gap in knowledge with respect to applying exogenous carbon monoxide as a treatment for clinical sepsis. *In vitro* experiments cannot fully mimic the pathophysiology of patients in clinical settings, and further studies are therefore required to explore the detailed pathophysiology of platelets during sepsis and to provide a more comprehensive understanding of the pharmacokinetics and biology of CO.

## Additional Information

**How to cite this article**: Liu, D. *et al.* Suppressive effect of exogenous carbon monoxide on endotoxin-stimulated platelet over-activation via the glycoprotein-mediated PI3K-Akt-GSK3β pathway. *Sci. Rep.* **6**, 23653; doi: 10.1038/srep23653 (2016).

## Supplementary Material

Supplementary Information
