# Supplementary material for: Suppressive effect of exogenous carbon monoxide on endotoxin-stimulated platelet over-activation via the glycoprotein-mediated PI3K-Akt-GSK3β pathway
Source: Sci Rep. 2016 Mar 29;6:23653. doi: 10.1038/srep23653 (PMC4810323; doi:10.1038/srep23653)
Supplement: Supplementary Information [file srep23653-s1.doc]

**Suppressive effect of exogenous carbon monoxide on endotoxin stimulated platelet over-activation via glycoprotein-mediated PI3K-Akt-GSK3β pathway**

**Running title:** Carbon monoxide suppresses platelet activation

Dadong Liu^1,*^, Xu Wang^2*^, Weiting Qin^2^, Jingjia Chen^2^, Yawei Wang^3^, Mingfeng Zhuang^2^, Bingwei Sun^2^

^1^ Department of Critical Care Medicine, Affiliated Hospital, JiangsuUniversity, Zhenjiang, Jiangsu Province, China

^2^ Department of Burns and Plastic Surgery, Affiliated Hospital, Jiangsu University, Zhenjiang, Jiangsu Province, China

^3^School of Science, Jiangsu University, Zhenjiang, Jiangsu Province, China

^*^ **These authors contributed equally to this work.**

**Correspondence to:** Professor Bingwei Sun, MD, PhD, Department of Burns and Plastic Surgery of Affiliated Hospital, Jiangsu University, Zhenjiang 212001, Jiangsu Province, China

**Telephone:** +86-511-85082258  **Fax:** +86-511-85029089

**Email:**[sunbinwe@hotmail.com](mailto:sunbinwe@hotmail.com)

**Supplementary Materials**

**Materials**

Tricarbonyldichlororuthenium (II) dimer (carbon monoxide releasing molecule-2, CORM-2, Chemical structure shown in Supplementary Fig 1) was obtained from Sigma Aldrichc (St Louis, Mo., USA) and solubilized in dimethyl sulfoxide (DMSO) to obtain a 40 mmol/L stock. Inactive form of CORM-2 (negative controls) was used in some experiments and prepared as follows: CORM-2 was inactivated (iCORM-2) by leaving the stock of CORM-2 at 37°C in a 5% CO_2_ humidified atmosphere for 24h to liberate CO[^1^](#_ENREF_1). The iCORM-2 solution was finally bubbled with nitrogen to remove the residual CO present in the solution. The FITC-labeled CD41 monoclonal antibody (mAb) and eFluor 660-labeled GPⅥ mAb were obtained from eBioscience (San Diego, CA, USA). Theβ-actin goat mAb, Phosphatase inhibitor cocktail, PE-labeled P-Selectin/GPIbα/integrin β_3_ mAb were from Santa Cruz Biotechnology (Dallas, Texas, USA). The GSK-3β (27C10) rabbit mAb, Phospho-GSK-3β(Ser9) rabbit mAb antibody, Akt(pan) rabbit mAb, Phospho-Akt(Ser473) rabbit mAb antibody , LY294002 (PI3K inhibitor), and CHIR99021 (GSK-3β inhibitor) were from Cell Signaling Technology (Boston, MA, USA). The cGMP Elisa kit was from R&D Systems (Minneapolis, MN, USA). The anti-PI3-Kinase rabbit antibody p110β and Western HRP substrate were from Merck Millipore (Billerica, MA, USA). SH-6 (Akt inhibitor) was obtained from BioVision Corporation（Milpitas, CA, USA）.The FACS Calibur BD Flow Cytometer was from Becton Dickison Corporation (San Jose, CA, USA). The CO_2_ incubator (Napco 5400) and automatic coagulation analyzer were from Beckman Coulter Corporation. Silicon Nitride probes was obtained from Applied NanoStructures, Inc (Mountain View, CA, USA). Atomic force microscopy (AFM) was obtained from Asylum Research (Santa Barbara, CA, USA). All the other chemicals were of reagent grade and obtained from Sigma unless otherwise stated.

**Ethics Statement**

The Medical Ethical Committee of Jiangsu University approved the study. After written informed consent which is in accordance with the Declaration of Helsinki, blood specimens were extracted from healthy drug-free donors’ cubital veins. Consent for the use of these samples was given by the Medical Ethical Committee of Jiangsu University.

**Preparation of washed platelets**

Blood was obtained with approval from the Medical Ethical Committee of Jiangsu University from healthy drug-free volunteers. Blood was collected into the vacuum tube containing and anticoagulated with one-nine of 129 mM trisodium citrate. 30ml blood was obtained each times and 600ml of blood was consumed at end of this study. Platelet rich plasma (PRP) was obtained by centrifuging at 120×g for 10 min. Platelets were isolated by centrifuging at 678×g for 10 min and washed twice with CGS buffer. The platelet poor plasma (PPP) was used to measure the platelet aggregation. For experiments with PRP, the platelet concentration was maintained in 2×10^8^/mL at every experiment.

**LPS-stimulated platelet model**

LPS（10 μg/mL）was used to stimulate PRP to induce the LPS-stimulated platelet model. This final concentration of LPS was used according to the published results in our laboratory and others. CORM-2 and iCORM-2 with different concentrations were administrated as potential therapeutic agenda. The experimental PRP was assigned to five groups randomly. The control group didn’t undergo any treatment, whereas the LPS group received LPS (10 μg/mL) treatment for 30min, the CORM-2 group and iCORM-2 group underwent the same stimulation of LPS with immediate administration of CORM-2 (10 or 50 μM) and iCORM-2 (50 μM), respectively. Additional experiments with CORM-2 pre-conditioning or delayed treatment were also performed. Briefly, CORM-2 preconditioning for 30 min followed by LPS stimulation for an additional 30 min was as CORM-2 preconditioning model; LPS stimulation for 30 min followed by CORM-2 treatment for an additional 30 min was as CORM-2 delayed treatment model.

Signaling molecular inhibitors, LY294002 (PI3K inhibitor), SH-6 (Akt inhibitor) and CHIR99021 (GSK-3α/β inhibitor), were used in designed experiments and incubated with the platelets for 10 minutes before LPS stimulation in additional experiments.

Samples were incubated in CO_2_-incubator at 37℃, 95% humidity, and 5%CO_2_. After being intervened, the correlated indexes were detected.

**Platelet adhesion and aggregation**

A total of 1.5 mL of sample from each group was added into the spherical glass bottles. The bottles were rotated at the speed of 3 r/min. 1 mL of blood from pre-rotated and post-rotated samples were collected. Automatic coagulation analyzer was used to count the number of the platelets in the collected samples. The platelet adhesion ratio was computed through the formula:

Platelet adhesion rate (%) = ((pre-rotated platelet count-post-rotated count)/pre-rotated platelet count) ×100.

Platelet aggregation was measured in a platelet aggregometer as previously described[^4^](#_ENREF_4). The samples (300μL) were incubated with magnetic bars at 37°C with stirring. The ADP was applied to introduce the platelet aggregation. Five minutes of the data were recorded.

**Platelet secretion**

The Dense Granule secretion was determined by measuring adenosine triphosphate (ATP) release as previous described[^5^](#_ENREF_5). Briefly, PRP were incubated with LPS at 37°C in the presence or absence of CORM-2 (10, 50μM) for 30 minutes. To measure the srcretion of ATP in the supernatant, luciferin-luciferase reagent was added to PRP (final concentration of 100nM) for 3s before luminescence was measured for 10s using a luminometer. Data were expressed as relative ATP levels.

α-Granule secretion was assessed as P-selectin exposure and examined by flow cytometry as previous described[^6^](#_ENREF_6). Briefly, PRP were incubated with LPS at 37°C presence or absence of CORM-2 (10, 50μM) at 30 minutes. Samples were fixed with 1% paraformaldehyde for 15 min at room temperature. After washed once with PBS, the platelets were incubated with PE-labeled anti-P-selectin and FITC-labeled-anti-CD41 in the dark for 30 min at room temperature. IgG1-PE was applied as isotype control antibodies. P-selectin expression was analyed by a FACSCalibur flow cytometer (BD Biosciences, USA).

**Platelet spreading**

Platelet spreading on immobilized fibrinogen was performed as previous describes[^7^](#_ENREF_7). Glass [slide](javascript:void(0);)s were coated with 30μg/L fibrinogen in PBS at 4°C overnight. Washed platelets preincubated with LPS at 37°C in the presence or absence of CORM-2 for 30 minutes, were allowed to adhere and spread on fibrinogen-coated slides at 37°C for 90 minutes. After three washes with PBS, the platelets were fixed, permeabilized and stained with FITC-labeled phalloidin. Image of spread platelets stained with FITC-labeled phalloidin were captured with Olympus IX71 fluorescence microscope (Olympus, Japan), and platelet counting was quantified using the Image-Pro plus 6.0 software.

**Flow cytometry**

The samples from all the groups were collected and fixed in 1% paraformaldehyde for 15 min at room temperature. The fixed samples were washed three times by phosphate buffer. They were then incubated with FITC-labeled CD41 (CD41-FITC). The GPⅥ-eFluor 660, GPIbα-PE, P-selectin-PE and integrin β_3_-PE were independently added into the samples and IgG1-eFluor 660 and IgG1-PE were applied as isotype control antibodies. All samples were incubated in the dark for 30 min at room temperature, washed three times and then analyzed by flow cytometry[^8^](#_ENREF_8).

**Immunofluorescence**

Samples of all groups were collected and fixed in 1% paraformaldehyde for 15 min at room temperature. The fixed platelets were spinned at 800 g for 5 min and permeabilized using 0.3% Triton-X-100. Samples were washed and blocked for 2 hours in 1% BSA. After washing with PBS, platelets were incubated with the corresponding antibodies overnight. Then primary antibodies were removed and Alexa Fluor conjugated anti–mouse/anti–rabbit antibodies (1:250 dilutions) were applied for 2 hour at room temperature. For visualization of granule distribution, fluorescent microscopy was performed using an Olympus IX71 microscope (Olympus Japan). All images were processed using cellSens Standard 1.12 software.

**Electron Microscopy**

Platelet morphological evaluation was conducted with a scanning electron microscope (LEO 440, Leica and Zeiss Co., Cambridge, England) or transmission electron microscope (JEM-2100, Japan Electronics Co., Ltd.) as previous described[^9^](#_ENREF_9). Briefly, PRP were incubated with LPS at 37°C in the presence or absence of CORM-2 (10, 50mM) for 30 minutes. Washed platelets were incubated in 2.5% glutaraldehyde solution for 1h at room temperature and saved at 4°C until use. Each sample was rinsed 4 times in time intervals of 30 min to remove the unbound glutaraldehyde. Osmium tetraoxide (OsO_4_), dissolved in isoosmolarPBS with citrate was added to the sample to obtain final concentration of 1%. The platelets fixed by glutaraldehyde were incubated for 1h at room temperature. Unbound OsO_4_ was removed by washing. The platelets were dehydrated by acetone (50%, 60%, and 90%) to dry the samples. The final step was performed in 100% acetone 1h with two exchanges of acetone. Samples were dried with liquid CO_2_. Dried samples were sputtered with gold to be observed by a Cambridge Instruments S360 scanning electron microscope. All experiments were analyzed double-blind by 3 researchers.

**Atomic force microscopy (AFM) Imaging**

The stimulated platelets were prepared as describes above. AFM used in the experiment was to acquire the topographic and phase images of platelet and the roughness of cell membrane with a tapping mode in atmospheric environment. The procedure was repeated for five cells and each cell was scanned for at least three times. After imaging the whole cells, the adhesive force of cell membrane was further assayed by AFM in a force-modulate mode, following the force-distance curves were drawn. All force–distance experiments were performed at the same loading rate. Adhesion forces were obtained from the contact process between the probe tip and sample surface.

**Phase microscopy technique**

Platelet was also observed by BioPhase. BioPhase is a new imaging device based on Digital Wavefront Technology that performs real time cellular imaging using standard optical microscopes. When light waves travel through the transparent biological specimens, the phase of the transmitted wave will change in a manner dependent on properties of the specimens. The changes of phase could be obtained by phase microscopy technique, which reveals the information about intracellular and extracellular structures.In it the volume, shape and the surface roughness of the intracellular structures, mainly the organelles, were involved. Briefly, the stimulated platelets were prepared as described above. Then samples were fixed by mixing them with an equal volume of 1 % paraformaldehyde for 15min at room temperature. Finally the platelets were visualized on the optical microscope (OLYMPUS GX51) using the BioPhase imaging device. All images were processed using MATLAB (r2014a) software.

**ELISA**

Platelet cGMP accumulation was measured by using a standard ELISA kit as previous described. Brifely, platelets were hydrolyzed with cell lysis buffer. The lysates were centrifuged and cGMP in supernatants was measured using cGMP ELISA Kit according to the manufacture’s protocol.

**Immunoprecipitation and Western blot**

PRP were incubated with LPS at 37°C at presence or absence of CORM-2 (10, 50mM) for 30 min. The reaction was stopped by the addition of equal volumes of the RIPA buffer that contained protease inhibitor and phosphatase inhibitor cocktails. The lysates were incubated for 30 min on ice for completion of the lysis action. The lysate was divided into two groups. A part of the lysate was collected by immunoprecipitation for detection of target phosphorylated protein. Another was processed by immunoblotting for detection of target proteins.

For immunoprecipitation studies, platelet lysates were centrifuged at 12,000 g for 10 min at 4°C to remove insoluble material. The nonspecific binding proteins were cleared by incubating platelet lysates with the target antibody alone overnight at 4°C, and then rotated with protein A-Sepharose beads for 4 hours at 4°C. The mixture was washed twice with 1× lysis buffer, and the bound proteins were eluted with SDS sample buffer and subjected to Western blotting with the indicated antibodies as described below.

Samples (10 μg of protein) were subjected to electrophoresis on 12% SDS-polyacrylamide gels, with the use of the discontinuous systemand transferred onto nitrocellulose membranes, which were incubated with different dilutions of primary and secondary antibodies. The bands were visualized by the use of ECL reagentand Hyperfilm ECL (Amersham, Arlington Heights, IL, USA) asdescribed by the manufacturer. Films were scanned using aflatbed scanner and the bands were quantified using BasicQuantifier software (Bio Image, Ann Arbor, MI, USA).

**Statistical Analyses**

Performed as described in the manuscript.

**Supplementary Figures and Figure Legends:**

**
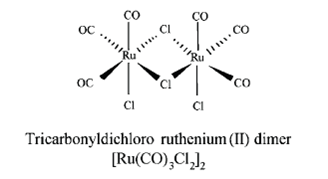
**

**Supplementary Figure 1.** Chemical structure of Tricarbonyldichlororuthenium (II) dimer (CORM-2).


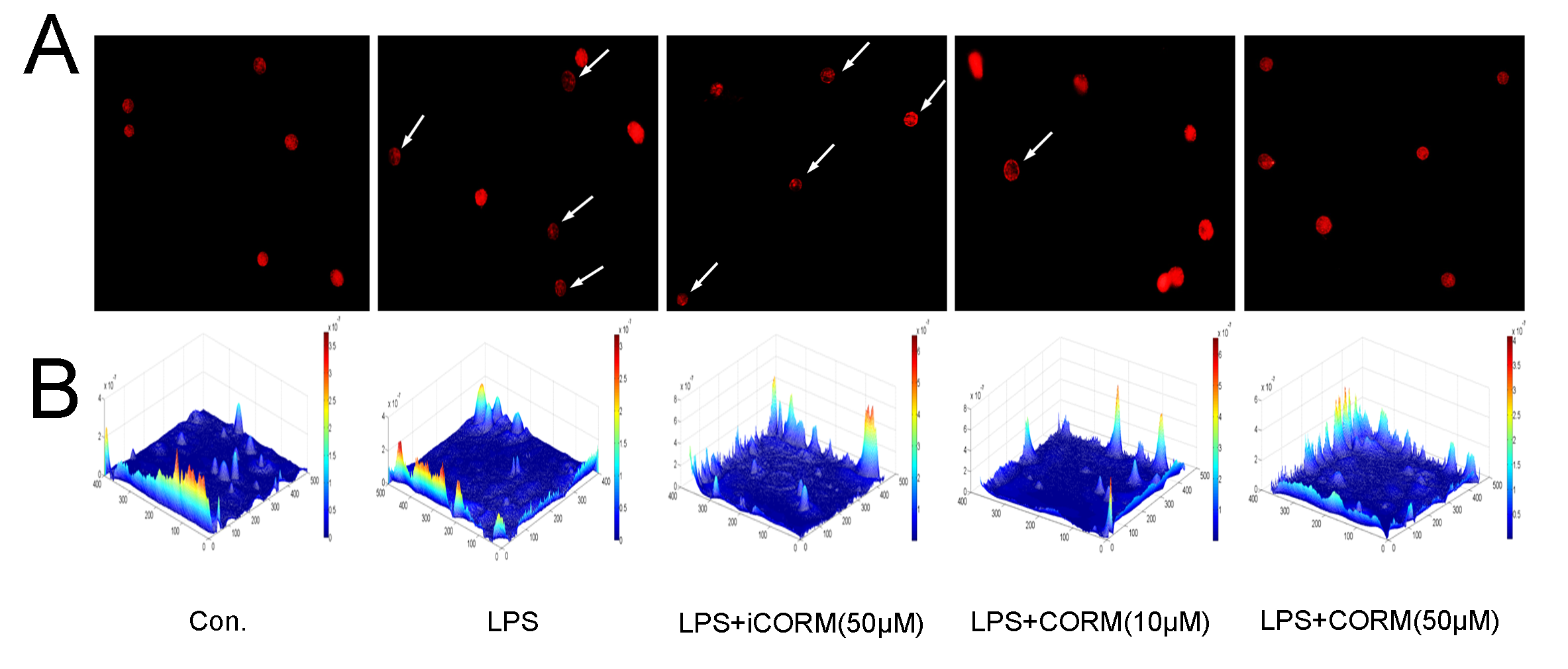


**Supplementary Figure 2. Effects of CORM-2 on the distribution of platelet α-granules and platelet phase shift.**

Platelets were stimulated by LPS and treated with CORM-2 as described in Fig. 1. All samples were collected, fixed, permeabilized and incubated with the VAMP8 antibodies overnight. Then, primary antibodies were removed and Alexa Fluor conjugated antibodies were applied. The fluorescent microscopy was performed to visualizate the **α-**granules distribution. The platelet α-granules were represented by the red fluorescence. The whole immunofluorescence images (A) showed that platelet α-granules moved from the central granulomere to the platelet plasma membrane. Inactivity CORM-2 intervention couldn’t alter the movement. Representative α-granules were indicated by the white arrow. Following CORM-2 treatment, the movement of platelet α-granules was significantly decreased. The 3D phase rendered images of platelets was shown in B. Images are corresponding to control, LPS, LPS+iCORM-2, LPS+CORM-2 (10μM), LPS+CORM-2 (50μM) group, respectively.

**
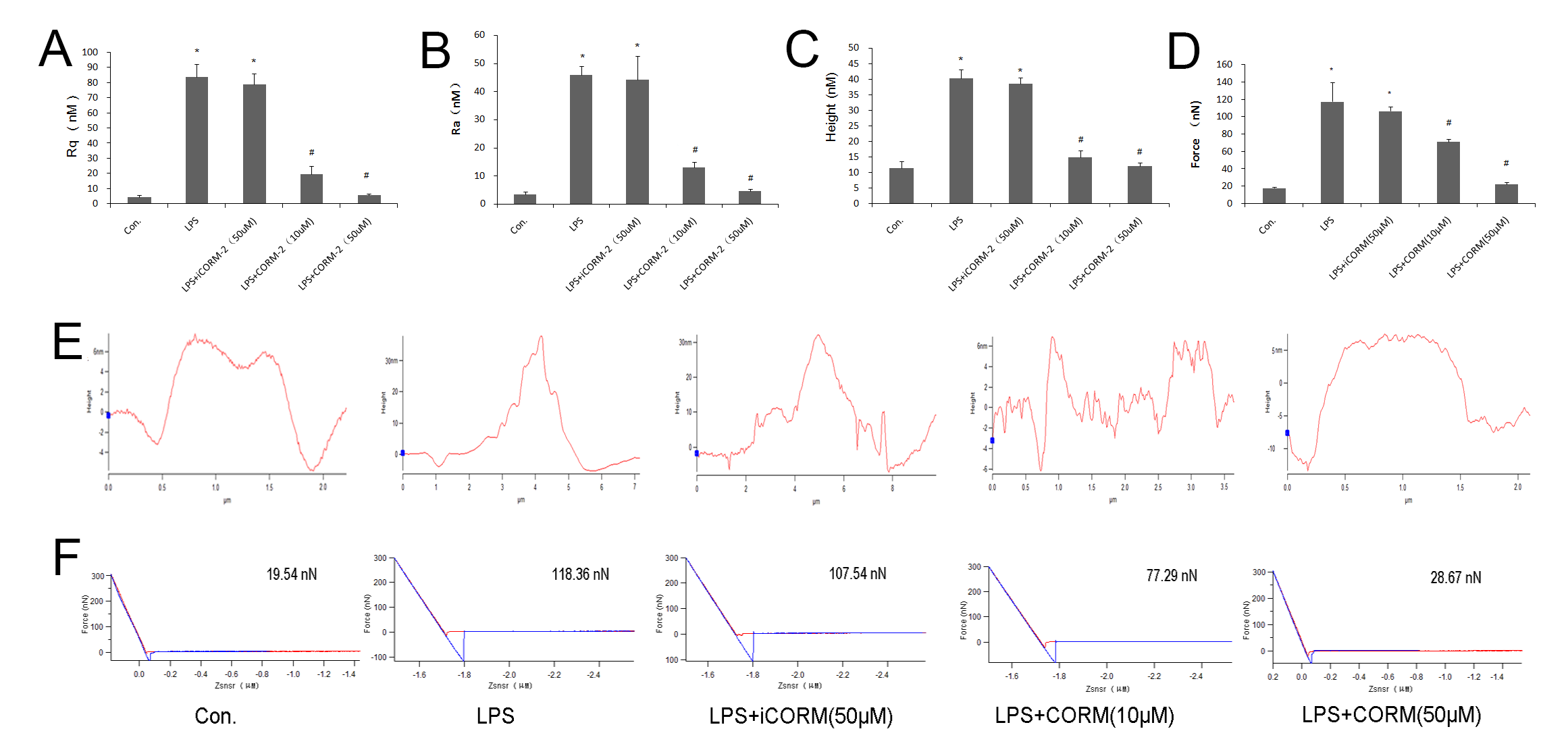
**

**Supplementary Figure 3. Effects of CORM-2 on platelet surface character in AFM.**

Platelets were stimulated by LPS and treated with CORM-2 as described in Fig. 1. All samples were collected and touched by a probe which was placed at the micro cantilever in the AFM. 1.08 μm area was selected from the platelets to calculate the Ra and Rq. As shown in A,B, results of control group indicated that the Rq and Ra values were 4.56 ± 1.03 and 3.29 ± 1.09 nm, respectively. The values were significantly increased in the LPS challenged platelets. Rq and Ra values were 83.81 ± 8.23 and 45.74 ± 3.17 nm. Nevertheless, the increase was inhibited in 10 μM CORM-2 group with the values of 19.46 ± 5.61 and 12.90 ± 2.10 nm. Besides, when treated with 50 μM CORM-2, the attenuation was more obvious with the values of 5.60 ± 0.91 and 4.64 ± 0.66 nm. The height profiles of platelets were observed in C and E. Results showed that after LPS stimulation, the height curve of platelet was manifested as a steep peak, meanwhile the average height was increased with the value of 40.16 ± 2.85 nm. However, co-incubation with 10 μM CORM-2 significantly reversed the height profile of platelet (14.84 ± 2.26 nm) and dual peaks with a rough curve were seen. The height profile was further improved when treated with 50 μM CORM-2 (12.04±0.98 nm). The force-distance information was acquired to indicate the adhesive force of cell membrane. As shown in D, F, in control group the adhesive force was 18.06 ± 0.99 nN. After LPS stimulation, the adhesive force was increased with the value of 117.48 ± 21.91 nN. while, this increase was attenuated when treated with CORM-2 in a dose dependent manner with the values of 71.10 ± 3.47 nN (10 μM) and 22.42±2.60 nN (50 μM), respectively. n=5 for each group. Results are described as mean ± SE of five experiments, *P<0.01 as compared to control; ﹟P < 0.05 as compared to LPS.

**
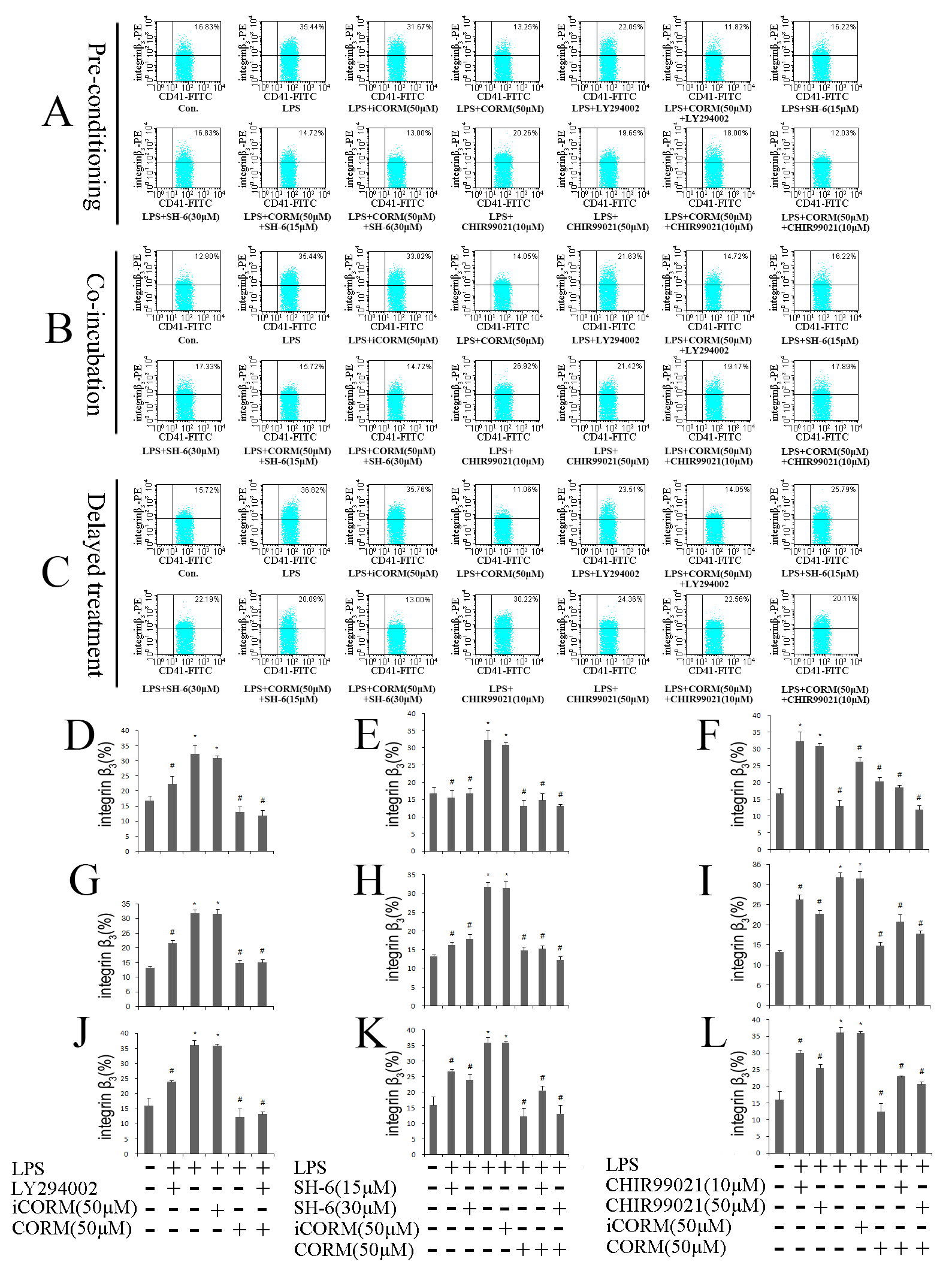
**

**Supplementary Figure 4. Effects of CORM-2 and signal molecular inhibitors on expression of integrinβ_3_ in** **LPS-stimulated platelet.** Platelets were stimulated by LPS and treated with CORM-2 as described in Fig. 1. All samples were incubated in the dark for 30 min, washed three times and analyzed by flow cytometry. Expressions of integrinβ_3_ were significantly upregulated in LPS-stimulated platelet, while exogenous CO and PI3K inhibitor (LY294002), Akt inhibitor (SH-6), GSK-3β inhibitor (CHIR99021) administration inhibited this upregulation(B,G,H,I). Similar results were also shown in CORM-2 pre-conditioning (A,D,E,F) and CORM-2 delayed treatment groups (C,J,K,L). Results are described as mean ± SE (n=5), *P<0.01 as compared to control; ﹟P < 0.05 as compared to LPS. Note that LPS-induced membrane glycoprotein expressions were down-regulated by CORM-2 in a dose-dependent manner.

**
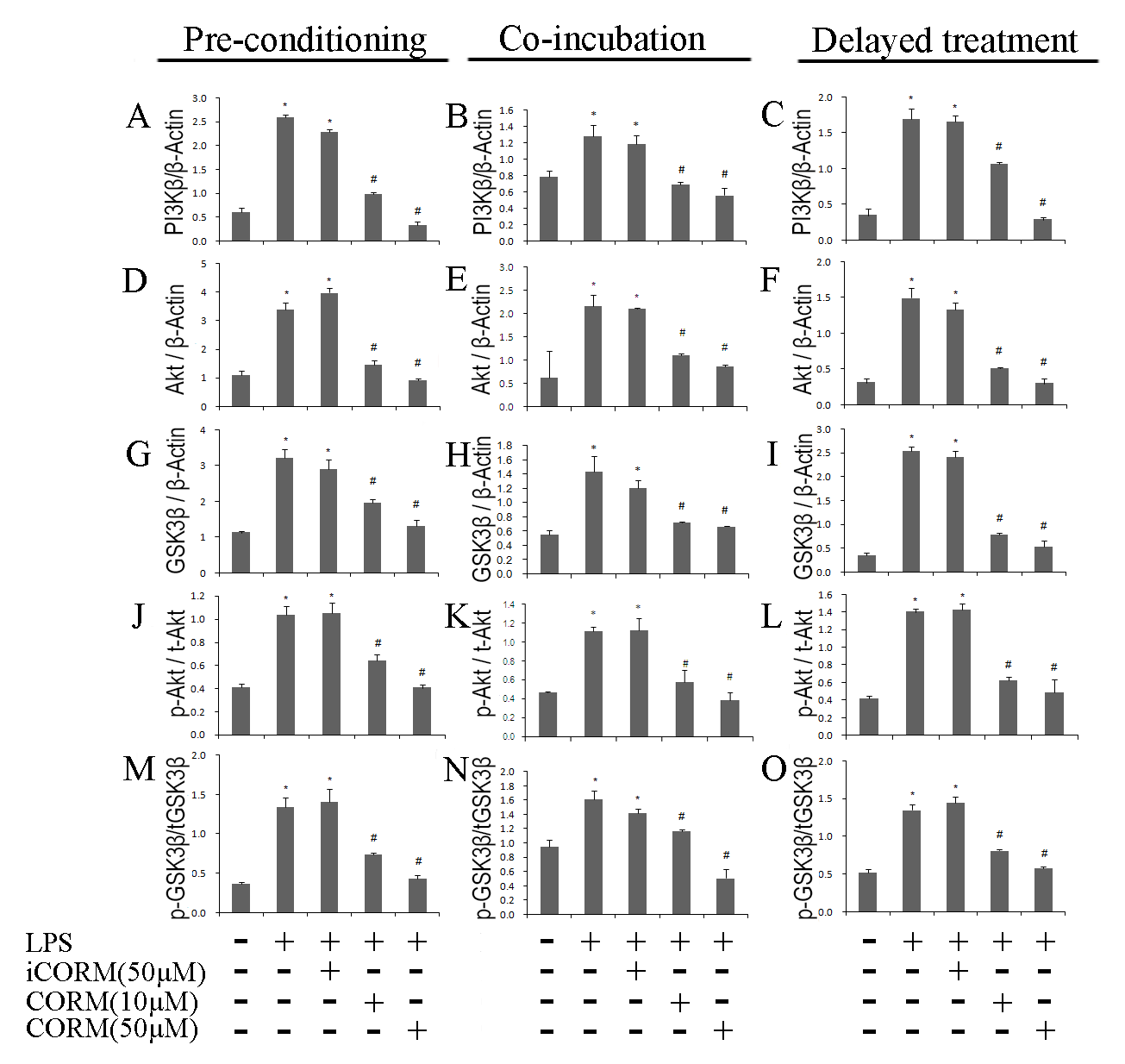
**

**Supplementary Figure 5. Effects of CORM-2 on signal molecular expression/ phosphorylation** in **LPS-stimulated platelet**. Platelets were stimulated by LPS and treated with CORM-2 as described in Fig. 1. Plateles were lysed in RIPA buffer that contained protease and phosphatase inhibitor cocktails. Signal molecular expression and phosphorylation were assessed by Western blot and immunoprecipitation. The bands were visualized and scanned by the use of ECL method and presented in the Figure 5 in the main text. Then, the bands were quantified using Basic Quantifier software and presented using histograms here. LPS stimulation resulted in a significant increase of PI3Kβ level. In contrast, this increase of PI3Kβ level was abolished in LPS-stimulated platelets treated with CORM-2 (B). Treatment of exogenous CO, Akt production (E) and phosphorylation in LPS-stimulated platelets were markedly inhibited (K). The same results appear in GSK-3β production and phosphorylation(H,N). Similar results were also shown in CORM-2 pre-conditioning (A,D,G,J,M) and CORM-2 delayed treatment groups (C,F,I,L,O). The average ratio of protein expression and phosphorylation level are described as mean ± SE (n=5), *P<0.01 as compared to control; ﹟P < 0.05 as compared to LPS. Note that LPS-induced signal molecular expression and phosphorylation were down-regulated by CORM-2 in a dose-dependent manner.

**
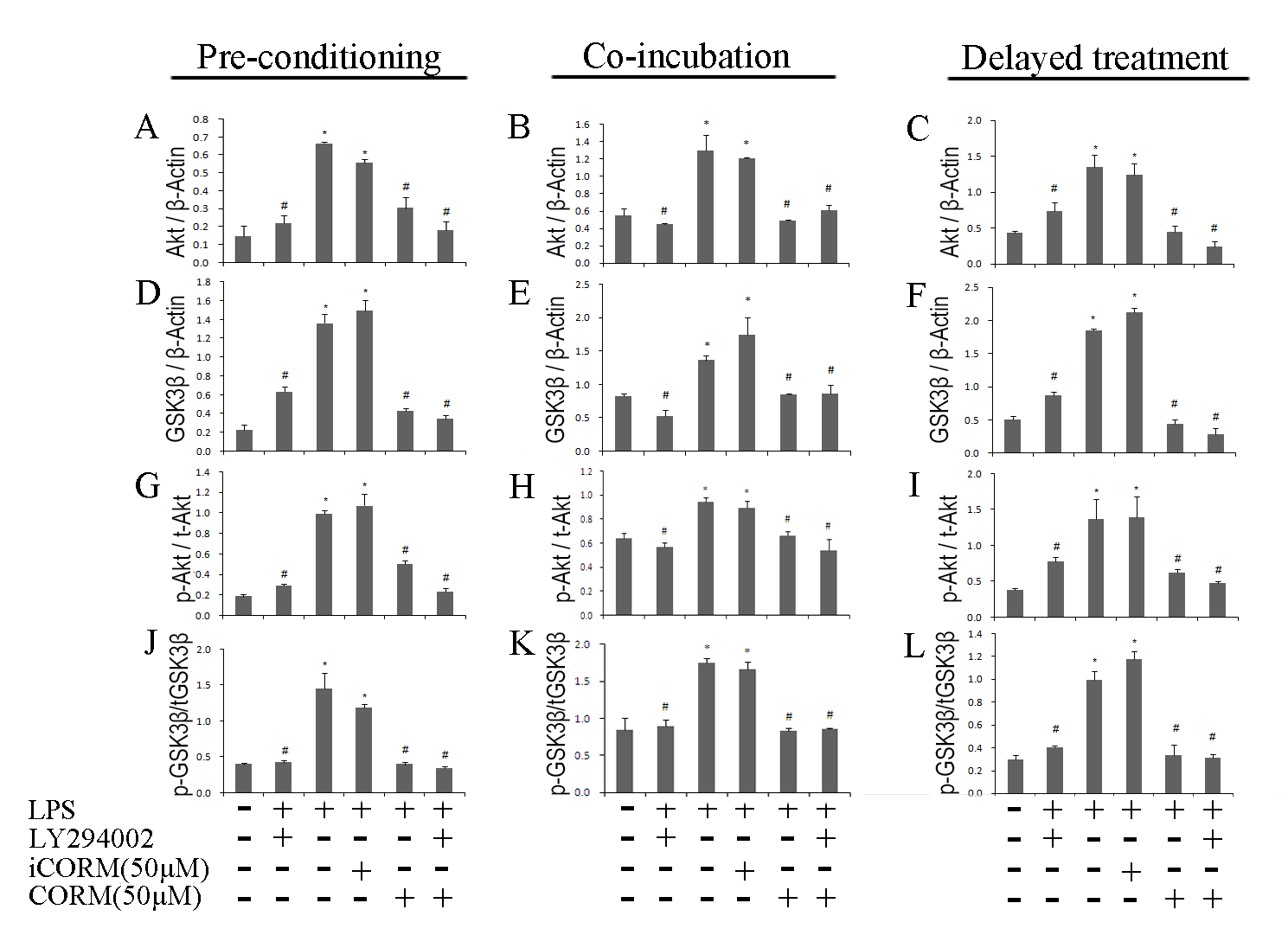
**

**Supplementary Figure 6. Effects of CORM-2 and PI3K inhibitor LY294002 in LPS-stimulated platelet.** Platelets were stimulated by LPS and treated with CORM-2 as described in Fig. 1. The platelets were lysed in RIPA buffer that contained protease and phosphatase inhibitor cocktails. Signal molecular expression and phosphorylation were assessed in SPD with treatment of CORM-2 or PI3K inhibitor LY294002. The increase of Akt production and phosphorylation were abolished simultaneously in LPS-stimulated platelet treated with LY294002 or CORM-2 (B,H). Meanwhile, GSK-3β expression and phosphorylation were inhibited in LPS-stimulated platelet with administration of LY294002 or CORM-2 (E,K), and no differences were observed between the LY294002 and CORM-2 (50 μM) group. Similar results were also shown in CORM-2 pre-conditioning (A,G,D,J) and CORM-2 delayed treatment groups (C,I,F,L). The average ratio of protein expression and phosphorylation level are described as mean ± SE (n=5) of three experiments, *P<0.01 as compared to control; ﹟P < 0.05 as compared to LPS.

**
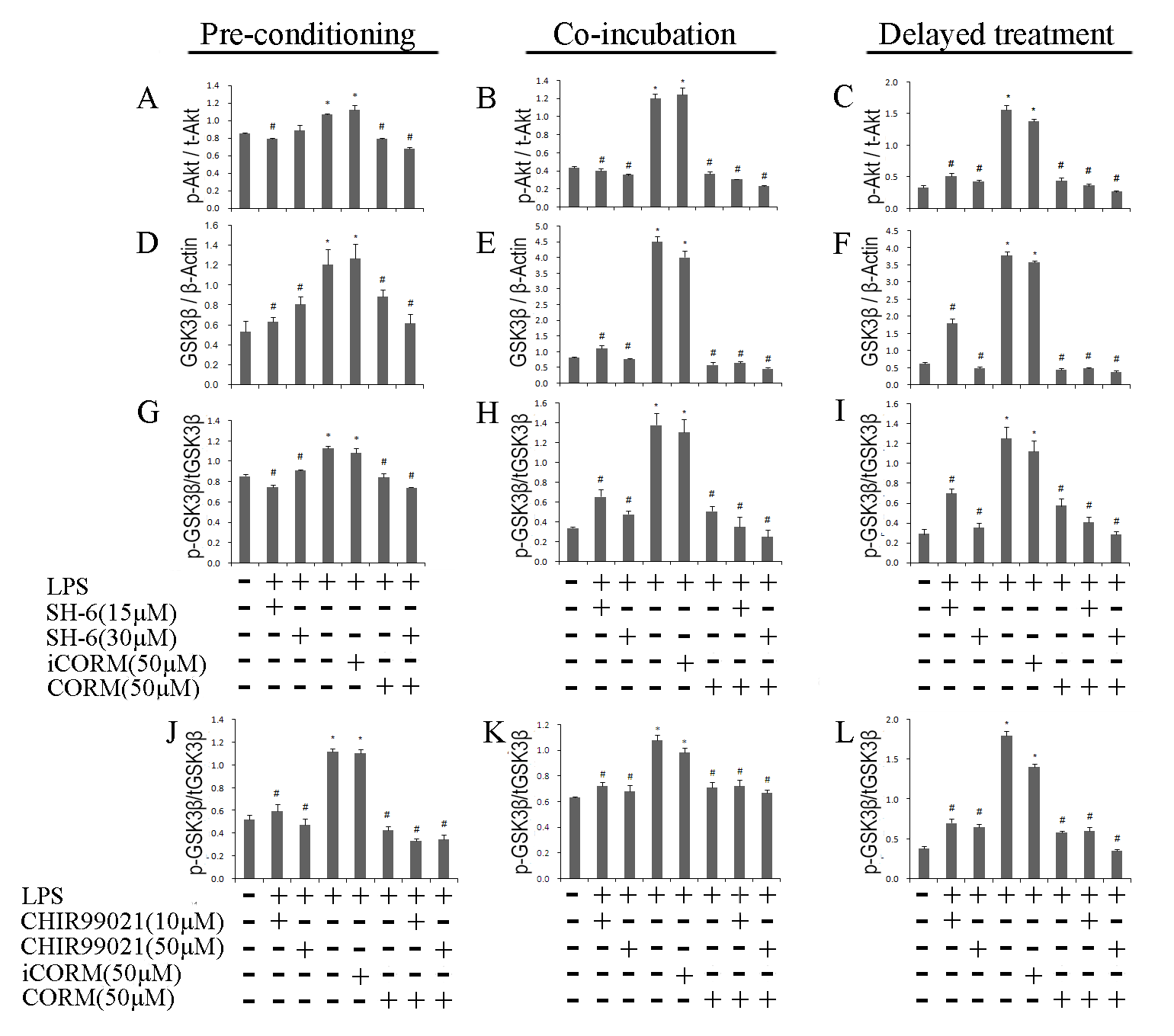
**

**Supplementary Figure 5. Effects of CORM-2 and Akt inhibitor SH-6 in SPD.** Platelets were stimulated by LPS and treated with CORM-2 as described in Fig. 1. The platelets were lysed in RIPA buffer that contained protease and phosphatase inhibitor cocktails. Signal molecular expression and phosphorylation were assessed in LPS-stimulated platelets with treatment of CORM-2 or Akt inhibitor SH-6. The increase of Akt production and phosphorylation were abolished simultaneously in LPS-stimulated platelet treated with SH-6 or CORM-2 (B). Meanwhile, GSK-3β expression and phosphorylation were inhibited in LPS-stimulated platelet with administration of SH-6 or CORM-2 (E,H), and no differences were observed between SH-6 and CORM-2 (50 μM) group. Similar results were also shown in CORM-2 pre-conditioning (A,D,G) and CORM-2 delayed treatment groups (C,F,I). In order to better clear the effect of GSK-3β on platelet activation, GSK-3β phosphorylation inhibitor (CHIR99021) were used in the further exploration.The increase of GSK-3β phosphorylation was inhibited in LPS-stimulated SPD treated with CHIR99021 (K). Similar results were also shown in CORM-2 pre-conditioning (J) or CORM-2 delayed treatment group (L). The average ratio of protein expression and phosphorylation level are described as mean ± SE (n=5) of three experiments, *P<0.01 as compared to control; ﹟P < 0.05 as compared to LPS.

**Supplemental References:**

1 Sun, B., Sun, Z., Jin, Q. & Chen, X. CO-releasing molecules (CORM-2)-liberated CO attenuates leukocytes infiltration in the renal tissue of thermally injured mice. *International journal of biological sciences* **4**, 176-183 (2008).

2 Sun, B., Zou, X., Chen, Y., Zhang, P. & Shi, G. Preconditioning of carbon monoxide releasing molecule-derived CO attenuates LPS-induced activation of HUVEC. *International journal of biological sciences* **4**, 270-278 (2008).

3 Zhang, G. *et al.* Lipopolysaccharide stimulates platelet secretion and potentiates platelet aggregation via TLR4/MyD88 and the cGMP-dependent protein kinase pathway. *Journal of immunology* **182**, 7997-8004, doi:10.4049/jimmunol.0802884 (2009).

4 Li, Z. *et al.* A stimulatory role for cGMP-dependent protein kinase in platelet activation. *Cell* **112**, 77-86 (2003).

5 Kahner, B. N. *et al.* Hematopoietic lineage cell specific protein 1 (HS1) is a functionally important signaling molecule in platelet activation. *Blood* **110**, 2449-2456, doi:10.1182/blood-2006-11-056069 (2007).

6 Trzeciak-Ryczek, A., Tokarz-Deptula, B. & Deptula, W. Platelets--an important element of the immune system. *Polish journal of veterinary sciences* **16**, 407-413 (2013).

7 Yin, H., Stojanovic, A., Hay, N. & Du, X. The role of Akt in the signaling pathway of the glycoprotein Ib-IX induced platelet activation. *Blood* **111**, 658-665, doi:10.1182/blood-2007-04-085514 (2008).

8 van Velzen, J. F., Laros-van Gorkom, B. A., Pop, G. A. & van Heerde, W. L. Multicolor flow cytometry for evaluation of platelet surface antigens and activation markers. *Thrombosis research* **130**, 92-98, doi:10.1016/j.thromres.2012.02.041 (2012).

9 Mrvar-Brecko, A. *et al.* Isolated microvesicles from peripheral blood and body fluids as observed by scanning electron microscope. *Blood cells, molecules & diseases* **44**, 307-312, doi:10.1016/j.bcmd.2010.02.003 (2010).
